# Supplementary material for: HIVprotI: an integrated web based platform for prediction and design of HIV proteins inhibitors
Source: J Cheminform. 2018 Mar 9;10:12. doi: 10.1186/s13321-018-0266-y (PMC5845081; doi:10.1186/s13321-018-0266-y)
Supplement: Supplementary file 2 — Additional file 2. Source code of HIVProtI web server. [file 13321_2018_266_MOESM2_ESM.zip › HIVprotI_Source-code/hivprotI/weka-3-6-6/doc/weka/filters/unsupervised/instance/RemoveMisclassified.html]

RemoveMisclassified


---


|  |  |  |
| --- | --- | --- |
| **Overview** \| **Package** \| **Class** \| **Tree** \| **Deprecated** \| **Index** \| **Help** \| **Weka's home** | |  |
| **PREV CLASS**   **NEXT CLASS** | **FRAMES**    **NO FRAMES**     **All Classes** |  |
| SUMMARY: NESTED \| FIELD \| CONSTR \| METHOD | DETAIL: FIELD \| CONSTR \| METHOD |  |


---


## weka.filters.unsupervised.instance Class RemoveMisclassified

```
java.lang.Object
  weka.filters.Filter
      weka.filters.unsupervised.instance.RemoveMisclassified
```

**All Implemented Interfaces:**: java.io.Serializable, CapabilitiesHandler, OptionHandler, RevisionHandler, UnsupervisedFilter

---

``` public class RemoveMisclassified extends Filter implements UnsupervisedFilter, OptionHandler ```

A filter that removes instances which are incorrectly classified. Useful for removing outliers.


Valid options are:

```
 -W <classifier specification>
  Full class name of classifier to use, followed
  by scheme options. eg:
   "weka.classifiers.bayes.NaiveBayes -D"
  (default: weka.classifiers.rules.ZeroR)
```

```
 -C <class index>
  Attribute on which misclassifications are based.
  If < 0 will use any current set class or default to the last attribute.
```

```
 -F <number of folds>
  The number of folds to use for cross-validation cleansing.
  (<2 = no cross-validation - default).
```

```
 -T <threshold>
  Threshold for the max error when predicting numeric class.
  (Value should be >= 0, default = 0.1).
```

```
 -I
  The maximum number of cleansing iterations to perform.
  (<1 = until fully cleansed - default)
```

```
 -V
  Invert the match so that correctly classified instances are discarded.
```

**Version:**
:   $Revision: 5548 $

**Author:**
:   Richard Kirkby (rkirkby@cs.waikato.ac.nz), Malcolm Ware (mfw4@cs.waikato.ac.nz)

**See Also:**: Serialized Form

---

**Constructor Summary**

`RemoveMisclassified()`


| **Method Summary** | |
| --- | --- |
| `boolean` | `batchFinished()`             Signify that this batch of input to the filter is finished. |
| `java.lang.String` | `classifierTipText()`             Returns the tip text for this property |
| `java.lang.String` | `classIndexTipText()`             Returns the tip text for this property |
| `Capabilities` | `getCapabilities()`             Returns the Capabilities of this filter. |
| `Classifier` | `getClassifier()`             Gets the classifier used by the filter. |
| `int` | `getClassIndex()`             Gets the attribute on which misclassifications are based. |
| `boolean` | `getInvert()`             Get whether selection is inverted. |
| `int` | `getMaxIterations()`             Gets the maximum number of cleansing iterations performed |
| `int` | `getNumFolds()`             Gets the number of cross-validation folds used by the filter. |
| `java.lang.String[]` | `getOptions()`             Gets the current settings of the filter. |
| `java.lang.String` | `getRevision()`             Returns the revision string. |
| `double` | `getThreshold()`             Gets the threshold for the max error when predicting a numeric class. |
| `java.lang.String` | `globalInfo()`             Returns a string describing this filter |
| `boolean` | `input(Instance instance)`             Input an instance for filtering. |
| `java.lang.String` | `invertTipText()`             Returns the tip text for this property |
| `java.util.Enumeration` | `listOptions()`             Returns an enumeration describing the available options. |
| `static void` | `main(java.lang.String[] argv)`             Main method for testing this class. |
| `java.lang.String` | `maxIterationsTipText()`             Returns the tip text for this property |
| `java.lang.String` | `numFoldsTipText()`             Returns the tip text for this property |
| `void` | `setClassifier(Classifier classifier)`             Sets the classifier to classify instances with. |
| `void` | `setClassIndex(int classIndex)`             Sets the attribute on which misclassifications are based. |
| `boolean` | `setInputFormat(Instances instanceInfo)`             Sets the format of the input instances. |
| `void` | `setInvert(boolean invert)`             Set whether selection is inverted. |
| `void` | `setMaxIterations(int iterations)`             Sets the maximum number of cleansing iterations to perform - < 1 means go until fully cleansed |
| `void` | `setNumFolds(int numOfFolds)`             Sets the number of cross-validation folds to use - < 2 means no cross-validation. |
| `void` | `setOptions(java.lang.String[] options)`             Parses a given list of options. |
| `void` | `setThreshold(double threshold)`             Sets the threshold for the max error when predicting a numeric class. |
| `java.lang.String` | `thresholdTipText()`             Returns the tip text for this property |

**Methods inherited from class weka.filters.Filter**

`batchFilterFile, filterFile, getCapabilities, getOutputFormat, isFirstBatchDone, isNewBatch, isOutputFormatDefined, makeCopies, makeCopy, numPendingOutput, output, outputPeek, toString, useFilter, wekaStaticWrapper`

**Methods inherited from class java.lang.Object**

`equals, getClass, hashCode, notify, notifyAll, wait, wait, wait`

**Constructor Detail**

### RemoveMisclassified

```
public RemoveMisclassified()
```


**Method Detail**

### getCapabilities

```
public Capabilities getCapabilities()
```

:   Returns the Capabilities of this filter.

    :   **Specified by:**: `getCapabilities` in interface `CapabilitiesHandler` **Overrides:**: `getCapabilities` in class `Filter`
    :   **Returns:**: the capabilities of this object **See Also:**: `Capabilities`

---


### setInputFormat

```
public boolean setInputFormat(Instances instanceInfo)
                       throws java.lang.Exception
```

:   Sets the format of the input instances.

    :   **Overrides:**: `setInputFormat` in class `Filter`
    :   **Parameters:**: `instanceInfo` - an Instances object containing the input instance structure (any instances contained in the object are ignored - only the structure is required). **Returns:**: true if the outputFormat may be collected immediately **Throws:**: `java.lang.Exception` - if the inputFormat can't be set successfully

---


### input

```
public boolean input(Instance instance)
              throws java.lang.Exception
```

:   Input an instance for filtering.

    :   **Overrides:**: `input` in class `Filter`
    :   **Parameters:**: `instance` - the input instance **Returns:**: true if the filtered instance may now be collected with output(). **Throws:**: `java.lang.NullPointerException` - if the input format has not been defined.: `java.lang.Exception` - if the input instance was not of the correct format or if there was a problem with the filtering.

---


### batchFinished

```
public boolean batchFinished()
                      throws java.lang.Exception
```

:   Signify that this batch of input to the filter is finished.

    :   **Overrides:**: `batchFinished` in class `Filter`
    :   **Returns:**: true if there are instances pending output **Throws:**: `java.lang.IllegalStateException` - if no input structure has been defined: `java.lang.NullPointerException` - if no input structure has been defined,: `java.lang.Exception` - if there was a problem finishing the batch.

---


### listOptions

```
public java.util.Enumeration listOptions()
```

:   Returns an enumeration describing the available options.

    :   **Specified by:**: `listOptions` in interface `OptionHandler`
    :   **Returns:**: an enumeration of all the available options.

---


### setOptions

```
public void setOptions(java.lang.String[] options)
                throws java.lang.Exception
```

:   Parses a given list of options.


    Valid options are:

    ```
     -W <classifier specification>
      Full class name of classifier to use, followed
      by scheme options. eg:
       "weka.classifiers.bayes.NaiveBayes -D"
      (default: weka.classifiers.rules.ZeroR)
    ```

    ```
     -C <class index>
      Attribute on which misclassifications are based.
      If < 0 will use any current set class or default to the last attribute.
    ```

    ```
     -F <number of folds>
      The number of folds to use for cross-validation cleansing.
      (<2 = no cross-validation - default).
    ```

    ```
     -T <threshold>
      Threshold for the max error when predicting numeric class.
      (Value should be >= 0, default = 0.1).
    ```

    ```
     -I
      The maximum number of cleansing iterations to perform.
      (<1 = until fully cleansed - default)
    ```

    ```
     -V
      Invert the match so that correctly classified instances are discarded.
    ```

    :   **Specified by:**: `setOptions` in interface `OptionHandler`
    :   **Parameters:**: `options` - the list of options as an array of strings **Throws:**: `java.lang.Exception` - if an option is not supported

---


### getOptions

```
public java.lang.String[] getOptions()
```

:   Gets the current settings of the filter.

    :   **Specified by:**: `getOptions` in interface `OptionHandler`
    :   **Returns:**: an array of strings suitable for passing to setOptions

---


### globalInfo

```
public java.lang.String globalInfo()
```

:   Returns a string describing this filter

    :   **Returns:**: a description of the filter suitable for displaying in the explorer/experimenter gui

---


### classifierTipText

```
public java.lang.String classifierTipText()
```

:   Returns the tip text for this property

    :   **Returns:**: tip text for this property suitable for displaying in the explorer/experimenter gui

---


### setClassifier

```
public void setClassifier(Classifier classifier)
```

:   Sets the classifier to classify instances with.

    :   **Parameters:**: `classifier` - The classifier to be used (with its options set).

---


### getClassifier

```
public Classifier getClassifier()
```

:   Gets the classifier used by the filter.

    :   **Returns:**: The classifier to be used.

---


### classIndexTipText

```
public java.lang.String classIndexTipText()
```

:   Returns the tip text for this property

    :   **Returns:**: tip text for this property suitable for displaying in the explorer/experimenter gui

---


### setClassIndex

```
public void setClassIndex(int classIndex)
```

:   Sets the attribute on which misclassifications are based.
    If < 0 will use any current set class or default to the last attribute.

    :   **Parameters:**: `classIndex` - the class index.

---


### getClassIndex

```
public int getClassIndex()
```

:   Gets the attribute on which misclassifications are based.

    :   **Returns:**: the class index.

---


### numFoldsTipText

```
public java.lang.String numFoldsTipText()
```

:   Returns the tip text for this property

    :   **Returns:**: tip text for this property suitable for displaying in the explorer/experimenter gui

---


### setNumFolds

```
public void setNumFolds(int numOfFolds)
```

:   Sets the number of cross-validation folds to use
    - < 2 means no cross-validation.

    :   **Parameters:**: `numOfFolds` - the number of folds.

---


### getNumFolds

```
public int getNumFolds()
```

:   Gets the number of cross-validation folds used by the filter.

    :   **Returns:**: the number of folds.

---


### thresholdTipText

```
public java.lang.String thresholdTipText()
```

:   Returns the tip text for this property

    :   **Returns:**: tip text for this property suitable for displaying in the explorer/experimenter gui

---


### setThreshold

```
public void setThreshold(double threshold)
```

:   Sets the threshold for the max error when predicting a numeric class.
    The value should be >= 0.

    :   **Parameters:**: `threshold` - the numeric theshold.

---


### getThreshold

```
public double getThreshold()
```

:   Gets the threshold for the max error when predicting a numeric class.

    :   **Returns:**: the numeric threshold.

---


### maxIterationsTipText

```
public java.lang.String maxIterationsTipText()
```

:   Returns the tip text for this property

    :   **Returns:**: tip text for this property suitable for displaying in the explorer/experimenter gui

---


### setMaxIterations

```
public void setMaxIterations(int iterations)
```

:   Sets the maximum number of cleansing iterations to perform
    - < 1 means go until fully cleansed

    :   **Parameters:**: `iterations` - the maximum number of iterations.

---


### getMaxIterations

```
public int getMaxIterations()
```

:   Gets the maximum number of cleansing iterations performed

    :   **Returns:**: the maximum number of iterations.

---


### invertTipText

```
public java.lang.String invertTipText()
```

:   Returns the tip text for this property

    :   **Returns:**: tip text for this property suitable for displaying in the explorer/experimenter gui

---


### setInvert

```
public void setInvert(boolean invert)
```

:   Set whether selection is inverted.

    :   **Parameters:**: `invert` - whether or not to invert selection.

---


### getInvert

```
public boolean getInvert()
```

:   Get whether selection is inverted.

    :   **Returns:**: whether or not selection is inverted.

---


### getRevision

```
public java.lang.String getRevision()
```

:   Returns the revision string.

    :   **Specified by:**: `getRevision` in interface `RevisionHandler` **Overrides:**: `getRevision` in class `Filter`
    :   **Returns:**: the revision

---


### main

```
public static void main(java.lang.String[] argv)
```

:   Main method for testing this class.

    :   **Parameters:**: `argv` - should contain arguments to the filter: use -h for help


---


|  |  |  |
| --- | --- | --- |
| **Overview** \| **Package** \| **Class** \| **Tree** \| **Deprecated** \| **Index** \| **Help** \| **Weka's home** | |  |
| **PREV CLASS**   **NEXT CLASS** | **FRAMES**    **NO FRAMES**     **All Classes** |  |
| SUMMARY: NESTED \| FIELD \| CONSTR \| METHOD | DETAIL: FIELD \| CONSTR \| METHOD |  |


---
